# Supplementary material for: Interferon-based agents for current and future viral respiratory infections: A scoping literature review of human studies
Source: PLOS Glob Public Health. 2022 Apr 6;2(4):e0000231. doi: 10.1371/journal.pgph.0000231 (PMC10022196; doi:10.1371/journal.pgph.0000231)
Supplement: S2 Table — (DOCX) [file pgph.0000231.s002.docx]

**SI Table 2: Search terms**

| Database | Search String |
| --- | --- |
| Embase | *(('Human respiratory syncytial virus'/exp OR 'SARS coronavirus'/exp OR 'coronavirus disease 2019'/exp OR 'Coronavirus infection'/exp OR 'Severe acute respiratory syndrome coronavirus 2'/exp OR ‘Middle East respiratory syndrome coronavirus'/exp OR 'influenza'/exp OR 'Human rhinovirus'/exp OR 'Rhinovirus infection'/exp OR 'asthma'/exp OR 'pneumonia'/exp OR 'chronic obstructive lung disease'/exp) OR  (Respiratory-syncytial-virus OR respiratory-syncitial-virus OR Respiratory-syncytial-pneumovirus OR Human-orthopneumovirus OR syncytial-respiratory-virus OR RSV OR severe-acute-respiratory-syndrome OR Coronavirus OR SARS-COV* OR COVID* OR 2019-nCoV OR nCoV-2019 OR MERS OR Influenza OR ‘Flu’ OR rhinovirus OR common-cold OR Asthma OR Pneumonia OR chronic-obstructive-lung-disease OR chronic-obstructive-lung-disorder OR chronic-obstructive-bronchitis OR chronic-obstructive-bronchopulmonary-disease OR chronic-obstructive-pulmonary-disease OR chronic-obstructive-pulmonary-disorder OR chronic-obstructive-respiratory-disease OR lung-chronic-obstructive-disease OR obstructive-lung-disease OR obstructive-lung-diseases OR obstructive-pulmonary-disease OR obstructive-respiratory-disease OR obstructive-respiratory-tract-disease OR COPD):ti,ab,kw)  AND   (('interferon'/exp OR 'toll like receptor'/exp OR 'toll like receptor 1'/exp OR 'toll like receptor 2'/exp OR 'toll like receptor 3'/exp OR 'toll like receptor 4'/exp OR 'toll like receptor 5'/exp OR 'toll like receptor 6'/exp OR 'toll like receptor 7'/exp OR 'toll like receptor 8'/exp OR 'toll like receptor 9'/exp OR 'toll like receptor 10'/exp OR 'ifn stimulated gene'/exp OR 'interferon stimulated gene'/exp OR 'interferon inducing agent'/exp OR 'toll like receptor agonist'/exp OR 'cridanimod'/exp OR 'avridine'/exp OR 'bendazol'/exp OR 'bropirimine'/exp OR 'imiquimod'/exp OR 'jacalin'/exp OR 'larifan'/exp OR 'polyadenylic polyuridylic acid'/exp OR 'polyinosinic polycytidylic acid'/exp OR 'carboxymethylcellulose polycytidylic polyinosinic acid polylysine'/exp OR 'krestin'/exp OR 'carboxyethylgermanium sesquioxide'/exp OR 'pyran copolymer'/exp OR 'ridostin'/exp OR 'nucleinate sodium'/exp OR 'thymostimulin'/exp OR 'tilorone'/exp OR 'tolpa'/exp) OR (Interferon* OR Toll-like-receptor* OR toll-like-receptor-1 OR toll-like-receptor-2 OR toll-like-receptor-3 OR toll-like-receptor-4 OR toll-like-receptor-5 OR toll-like-receptor-6 OR toll-like-receptor-7 OR toll-like-receptor-8 OR toll-like-receptor-9 OR toll-like-receptor-10 OR TLR1 OR TLR2 OR TLR3 OR TLR4 OR TLR5 OR TLR6 OR TLR7 OR TLR8 OR TLR9 OR TLR10 OR TLR-1 OR TLR-2 OR TLR-3 OR TLR-4 OR TLR-5 OR TLR-6 OR TLR-7 OR TLR-8 OR TLR-9 OR TLR-10 OR IFN-stimulated-gene* OR ISG OR IFN-stimulating-agent* OR IFN-induced-gene* OR ((inflammasome OR RIG-I-like-receptors OR retinoic-acid-inducible-gene-I-like-receptors OR RLR OR intracellular-RNA-sensor OR intracellular-DNA-sensor OR STING OR MAIVS) NEAR/3 agonist*) OR IFN-inducer* OR 10-carboxymethyl-9-acridanone OR avridine OR bendazol* OR bropirimine OR Imiquimod OR jacalin OR larifan OR Poly-A-U OR Poly-I-C OR polyinosinic-polycytidylic-acid OR poly-ICLC OR polysaccharide-K OR krestin OR crestine OR proxigermanium OR carboxyethylgermanium-sesquioxide OR Pyran-Copolymer OR divema OR ridostin OR sodium-nucleinate OR thymostimulin OR Tilorone OR Tolpa OR TLR-agonist* OR agatolimod OR baloramotide-plus-cadalimogene-ixalentivec-plus-glucopyranosyl-lipid-a OR baloramotide-plus-glucopyranosyl-lipid-a OR cobitolimod OR cpg-10101 OR entolimod OR epetirimod OR gardiquimod OR glucopyranosyl-lipid-A OR imiquimod OR isatoribine OR lapretolimod OR lefitolimod OR litenimod OR loxoribine OR motolimod OR resiquimod OR resiquimod-pegol OR rintatolimod OR selgantolimod OR sotirimod OR telratolimod OR vesatolimod):ti,ab,kw)  AND   (('inhalational drug administration'/lnk OR 'intranasal drug administration'/lnk OR 'inhalational drug administration'/exp OR 'intranasal drug administration'/exp) OR ((administration OR instillation OR medication OR therapy OR drug OR treatment OR dose) NEAR/3 (inhal* OR nasal OR intranasal OR intra-nasal OR nose)):ti,ab,kw)  AND   (('adverse drug reaction'/lnk OR 'complication'/lnk OR 'drug interaction'/lnk OR 'drug toxicity'/lnk OR 'side effect'/lnk OR 'unexpected outcome of drug treatment'/lnk OR 'lack of drug effect'/exp OR 'pharmacodynamics'/exp) OR (pharmacodynamics OR safety OR toxicity OR tolerability OR efficacy OR Drug-interaction OR Side-effect OR (adverse NEAR/2 reaction*) OR (lack NEAR/2 effect*)):ti,ab,kw)  AND   ('human'/exp OR 'human experiment'/exp OR 'patient'/exp OR patient*:ti,ab,kw)  AND   ('clinical trial'/exp OR 'clinical trial (topic)'/exp OR 'clinical trial topic'/exp OR 'adaptive clinical trial topic'/exp OR 'controlled clinical trial topic'/exp OR 'randomized controlled trial topic'/exp OR 'multicenter study topic'/exp OR 'phase 1 clinical trial topic'/exp OR 'phase 2 clinical trial topic'/exp OR 'phase 3 clinical trial topic'/exp OR 'phase 4 clinical trial topic'/exp)* |
| PubMed | *(("SARS Virus"[Mesh] OR "Severe Acute Respiratory Syndrome"[Mesh] OR "Coronavirus Infections"[Mesh]" OR COVID-19" [Supplementary Concept] OR "severe acute respiratory syndrome coronavirus 2" [Supplementary Concept]) OR (“Severe acute respiratory syndrome”[tiab] OR Coronavirus[tiab] OR SARS-COV[tiab] OR SARS-COV-1[tiab] OR COVID[tiab] OR COVID-19[tiab] OR COVID-2019[tiab] OR COVID19[tiab] OR SARS-CoV-2[tiab] OR 2019-nCoV[tiab] OR nCoV-2019[tiab]) OR (“Severe acute respiratory syndrome”[ot] OR Coronavirus[ot] OR SARS-COV[ot] OR SARS-COV-1[ot] OR COVID[ot] OR COVID-19[ot] OR COVID-2019[ot] OR COVID19[ot] OR SARS-CoV-2[ot] OR 2019-nCoV[ot] OR nCoV-2019[ot]))   AND   (("Interferons"[Mesh] OR "Toll-Like Receptors"[Mesh] OR "Interferon Inducers" [Pharmacological Action] OR "10-carboxymethyl-9-acridanone" [Supplementary Concept] OR "avridine" [Supplementary Concept] OR "bropirimine" [Supplementary Concept] OR "Imiquimod"[Mesh] OR "jacalin" [Supplementary Concept] OR "larifan" [Supplementary Concept] OR "Poly A-U"[Mesh] OR "Poly I-C"[Mesh] OR "poly ICLC" [Supplementary Concept] OR "poly IC-polylysine carboxymethyl dextran" [Supplementary Concept] OR "polysaccharide-K" [Supplementary Concept] OR "proxigermanium" [Supplementary Concept] OR "Pyran Copolymer"[Mesh] OR "ridostin" [Supplementary Concept] OR "sodium nucleinate" [Supplementary Concept] OR "thymostimulin" [Supplementary Concept] OR "Tilorone"[Mesh] OR "Tolpa" [Supplementary Concept] OR "CPG 10101" [Supplementary Concept] OR "CBLB502" [Supplementary Concept] OR "gardiquimod" [Supplementary Concept] OR "glucopyranosyl lipid-A" [Supplementary Concept] OR "TLR4 agonist G100" [Supplementary Concept] OR "isatoribine" [Supplementary Concept] OR "loxoribine" [Supplementary Concept] OR "VTX-2337" [Supplementary Concept] OR "resiquimod" [Supplementary Concept] OR "poly(I).poly(c12,U)" [Supplementary Concept] OR "vesatolimod" [Supplementary Concept]) OR (Interferon*[tiab] OR “Toll like receptor”[tiab] OR “Toll like receptors”[tiab] OR TLR[tiab] OR “IFN stimulated gene”[tiab] OR “IFN stimulated genes”[tiab] OR ISG[tiab] OR “IFN stimulating agent”[tiab] OR “IFN stimulating agents”[tiab] OR “IFN induced gene”[tiab] OR “IFN induced genes”[tiab] OR ((inflammasome[tiab] OR “RIG I like receptor” [tiab] OR “RIG I like receptors”[tiab] OR “retinoic acid inducible gene I like receptor”[tiab] OR “retinoic acid inducible gene I like receptors”[tiab] OR RLR[tiab] OR “intracellular RNA sensor”[tiab] OR “intracellular RNA sensors”[tiab] OR “intracellular DNA sensor”[tiab] OR “intracellular DNA sensors”[tiab] OR STING[tiab] OR MAIVS[tiab]) AND agonist*[tiab]) OR “IFN inducer”[tiab] OR “IFN inducers”[tiab] OR “10 carboxymethyl 9 acridanone”[tiab] OR avridine[tiab] OR bendazol*[tiab] OR bropirimine[tiab] OR Imiquimod[tiab] OR jacalin[tiab] OR larifan[tiab] OR Poly-A-U[tiab] OR Poly-I-C[tiab] OR “polyinosinic polycytidylic acid”[tiab] OR “poly ICLC”[tiab] OR “polysaccharide K”[tiab] OR krestin[tiab] OR crestine[tiab] OR proxigermanium[tiab] OR “carboxyethylgermanium sesquioxide”[tiab] OR “Pyran Copolymer”[tiab] OR divema[tiab] OR ridostin[tiab] OR “sodium nucleinate”[tiab] OR “nucleinate sodium”[tiab] OR thymostimulin[tiab] OR Tilorone[tiab] OR Tolpa[tiab] OR “TLR agonist”[tiab] OR “TLR agonists”[tiab] OR agatolimod[tiab] OR baloramotide[tiab] OR cobitolimod[tiab] OR “cpg 10101”[tiab] OR entolimod[tiab] OR epetirimod[tiab] OR gardiquimod[tiab] OR “glucopyranosyl lipid A”[tiab] OR imiquimod[tiab] OR isatoribine[tiab] OR lapretolimod[tiab] OR lefitolimod[tiab] OR litenimod[tiab] OR loxoribine[tiab] OR motolimod[tiab] OR resiquimod[tiab] OR rintatolimod[tiab] OR selgantolimod[tiab] OR sotirimod[tiab] OR telratolimod[tiab] OR vesatolimod[tiab]) OR (Interferon*[ot] OR “Toll like receptor”[ot] OR “Toll like receptors”[ot] OR TLR[ot] OR “IFN stimulated gene”[ot] OR “IFN stimulated genes”[ot] OR ISG[ot] OR “IFN stimulating agent”[ot] OR “IFN stimulating agents”[ot] OR “IFN induced gene”[ot] OR “IFN induced genes”[ot] OR ((inflammasome[ot] OR “RIG I like receptor” [ot] OR “RIG I like receptors”[ot] OR “retinoic acid inducible gene I like receptor”[ot] OR “retinoic acid inducible gene I like receptors”[ot] OR RLR[ot] OR “intracellular RNA sensor”[ot] OR “intracellular RNA sensors”[ot] OR “intracellular DNA sensor”[ot] OR “intracellular DNA sensors”[ot] OR STING[ot] OR MAIVS[ot]) AND agonist*[ot]) OR “IFN inducer”[ot] OR “IFN inducers”[ot] OR “10 carboxymethyl 9 acridanone”[ot] OR avridine[ot] OR bendazol*[ot] OR bropirimine[ot] OR Imiquimod[ot] OR jacalin[ot] OR larifan[ot] OR Poly-A-U[ot] OR Poly-I-C[ot] OR “polyinosinic polycytidylic acid”[ot] OR “poly ICLC”[ot] OR “polysaccharide K”[ot] OR krestin[ot] OR crestine[ot] OR proxigermanium[ot] OR “carboxyethylgermanium sesquioxide”[ot] OR “Pyran Copolymer”[ot] OR divema[ot] OR ridostin[ot] OR “sodium nucleinate”[ot] OR “nucleinate sodium”[ot] OR thymostimulin[ot] OR Tilorone[ot] OR Tolpa[ot] OR “TLR agonist”[ot] OR “TLR agonists”[ot] OR agatolimod[ot] OR baloramotide[ot] OR cobitolimod[ot] OR “cpg 10101”[ot] OR entolimod[ot] OR epetirimod[ot] OR gardiquimod[ot] OR “glucopyranosyl lipid A”[ot] OR imiquimod[ot] OR isatoribine[ot] OR lapretolimod[ot] OR lefitolimod[ot] OR litenimod[ot] OR loxoribine[ot] OR motolimod[ot] OR resiquimod[ot] OR rintatolimod[ot] OR selgantolimod[ot] OR sotirimod[ot] OR telratolimod[ot] OR vesatolimod[ot]))  AND   (("Administration, Inhalation"[Mesh] OR "Administration, Intranasal"[Mesh]) OR ((administration[tiab] OR instillation[tiab] OR medication[tiab] OR therapy[tiab] OR drug[tiab] OR treatment[tiab] OR dose[tiab]) AND (inhal*[tiab] OR nasal[tiab] OR intranasal[tiab] OR intra-nasal[tiab] OR nose[tiab] OR respiratory[tiab] OR aerosol[tiab])) OR ((administration[ot] OR instillation[ot] OR medication[ot] OR therapy[ot] OR drug[ot] OR treatment[ot] OR dose[ot]) AND (inhal*[ot] OR nasal[ot] OR intranasal[ot] OR intra-nasal[ot] OR nose[ot] OR respiratory[ot] OR aerosol[ot])))  AND   (("Drug-Related Side Effects and Adverse Reactions"[Mesh] OR "Drug Interactions"[Mesh]) OR  (Complications[tiab] OR pharmacodynamics[tiab] OR safety[tiab] OR toxicity[tiab] OR tolerability[tiab] OR efficacy[tiab] OR “Drug interaction”[tiab] OR “Drug interactions”[tiab] OR “Side effect”[tiab] OR “side effects”[tiab] OR “adverse reaction”[tiab] OR “adverse reactions”[tiab] OR “adverse drug reaction”[tiab] OR “adverse drug reactions”[tiab] OR “lack of effect”[tiab] OR “lack of drug effect”[tiab) OR (Complications[ot] OR pharmacodynamics[ot] OR safety[ot] OR toxicity[ot] OR tolerability[ot] OR efficacy[ot] OR “Drug interaction”[ot] OR “Drug interactions”[ot] OR “Side effect”[ot] OR “side effects”[ot] OR “adverse reaction”[ot] OR “adverse reactions”[ot] OR “adverse drug reaction”[ot] OR “adverse drug reactions”[ot]))  AND  (humans[sb] OR humans[mesh] OR "Patients"[Mesh] OR patient*[tiab] OR patient*[ot])  AND  (Clinical Trial[sb] OR Multicenter Study[sb] OR "Clinical Trials as Topic"[Mesh] OR "Multicenter Studies as Topic"[Mesh])* |
| Cochrane Library | *(MeSH descriptor: [SARS Virus] explode all trees OR MeSH descriptor: [Severe Acute Respiratory Syndrome] explode all trees OR MeSH descriptor: [Coronavirus Infections] explode all trees OR (“Severe acute respiratory syndrome” OR Coronavirus OR SARS-COV* OR COVID OR COVID-19 OR COVID-2019 OR COVID19 OR nCoV-2019 OR "novel coronavirus"))  AND  (MeSH descriptor: [Interferons] explode all trees OR MeSH descriptor: [Toll-Like Receptors] explode all trees OR MeSH descriptor: [Interferon Inducers] explode all trees OR MeSH descriptor: [Imiquimod] explode all trees OR MeSH descriptor: [Poly A-U] explode all trees OR MeSH descriptor: [Poly I-C] explode all trees OR MeSH descriptor: [Pyran Copolymer] explode all trees OR MeSH descriptor: [Tilorone] explode all trees OR (Interferon* OR “Toll like receptor” OR “Toll like receptors” OR TLR OR “IFN stimulated gene” OR “IFN stimulated genes” OR ISG OR “IFN stimulating agent” OR “IFN stimulating agents” OR “IFN induced gene” OR “IFN induced genes”) OR (((inflammasome OR “RIG I like receptor” OR “RIG I like receptors” OR “retinoic acid inducible gene I like receptor” OR “retinoic acid inducible gene I like receptors” OR RLR OR “intracellular RNA sensor” OR “intracellular RNA sensors” OR “intracellular DNA sensor” OR “intracellular DNA sensors” OR STING OR MAIVS) AND agonist*)) OR (“IFN inducer” OR “IFN inducers” OR “10 carboxymethyl 9 acridanone” OR avridine OR bendazol* OR bropirimine OR Imiquimod OR jacalin OR larifan OR Poly-A-U OR Poly-I-C OR “polyinosinic polycytidylic acid” OR “poly ICLC” OR “polysaccharide K” OR krestin OR crestine OR proxigermanium OR “carboxyethylgermanium sesquioxide” OR “Pyran Copolymer” OR divema OR ridostin OR “sodium nucleinate” OR “nucleinate sodium” OR thymostimulin OR Tilorone OR Tolpa OR “TLR agonist” OR “TLR agonists” OR agatolimod OR baloramotide OR cobitolimod OR “cpg 10101” OR entolimod OR epetirimod OR gardiquimod OR “glucopyranosyl lipid A” OR imiquimod OR isatoribine OR lapretolimod OR lefitolimod OR litenimod OR loxoribine OR motolimod OR resiquimod OR rintatolimod OR selgantolimod OR sotirimod OR telratolimod OR vesatolimod))  AND  (MeSH descriptor: [Administration, Inhalation] explode all trees OR MeSH descriptor: [Administration, Intranasal] explode all trees OR ((administration OR instillation OR medication OR therapy OR drug OR treatment OR dose) AND (inhal* OR nasal OR intranasal OR intra-nasal OR nose OR respiratory OR aerosol))  AND  (MeSH descriptor: [Drug-Related Side Effects and Adverse Reactions] explode all trees OR MeSH descriptor: [Drug Interactions] explode all trees OR (Complications OR pharmacodynamics OR safety OR toxicity OR tolerability OR efficacy OR “Drug interaction” OR “Drug interactions” OR “Side effect” OR “side effects” OR “adverse reaction” OR “adverse reactions” OR “adverse drug reaction” OR “adverse drug reactions” OR “lack of effect” OR “lack of effects” OR “lack of drug effect” OR “lack of drug effects”))  AND  Limit: In Trials* |
| medRxiv | *"SARS virus", "[Ss]evere acute respiratory syndrome", "[Cc]oronavirus", "SARS-COV", "COVID", "\\bn[Cc]o[Vv]\\b", "n-[Cc]o[Vv ]\\b", "novel coronavirus"  AND  "[Ii]nterferon", "[Tt]oll like receptor", "\\bTLR", "IFN stimulated gene", "\\bISG", "IFN stimulating agent", "IFN induced gene", "inflammasome", "RIG I like receptor", "retinoic acid inducible gene I like receptor ", "\\bRLR", "intracellular RNA sensor", "intracellular DNA sensor", "STING", "MAIVS", "IFN inducer", "10 carboxymethyl 9 acridanone", "[Aa]vridine", "[Bb]endazol", "[Bb]ropirimine", "[Ii]miquimod", "[Jj]acalin", "[Ll]arifan", "[Pp]oly-A-U", "[Pp]oly-I-C", "[Pp]olyinosinic [Pp]olycytidylic [Aa]cid", "[Pp]oly ICLC", "[Pp]olysaccharide K", "[Kk]restin", "[Cc]restine", "[Pp]roxigermanium", "[Cc]arboxyethylgermanium [Ss]esquioxide", "[Pp]yran [Cc]opolymer", "[Dd]ivema", "DIVEMA", "[Rr]idostin", "sodium nucleinate", "nucleinate sodium", "[Tt]hymostimulin", "[Tt]ilorone", "[Tt]olpa", "\\bTLR agonist", "[Aa]gatolimod", "[Bb]aloramotide", "[Cc]obitolimod", "cpg 10101", "CPG 10101", "[Ee]ntolimod", "[Ee]petirimod", "[Gg]ardiquimod", "glucopyranosyl lipid A\\b", "[Ii]satoribine", "[Ll]apretolimod", "[Ll]efitolimod", "[Ll]itenimod", "[Ll]oxoribine", "[Mm]otolimod", "[Rr]esiquimod", "[Rr]intatolimod", "[Ss]elgantolimod", "[Ss]otirimod", "[Tt]elratolimod", "[Vv]esatolimod"  AND  "inhal", "nasal", "\\bnose", "aerosol"  AND  "complications", "pharmacodynamics", "safety", "toxicity", "tolerability", "efficacy", "[Dd]rug interaction", "[Ss]ide effect", "[Aa]dverse reaction", "[Aa]dverse drug reaction", "lack of effect", "lack of drug effect", ""*  *Note –we included observational studies from mexRxiv* |
