# Supplementary material for: Interferon-based agents for current and future viral respiratory infections: A scoping literature review of human studies
Source: PLOS Glob Public Health. 2022 Apr 6;2(4):e0000231. doi: 10.1371/journal.pgph.0000231 (PMC10022196; doi:10.1371/journal.pgph.0000231)
Supplement: S3 Table — (DOCX) [file pgph.0000231.s003.docx]

**SI Table 3: Study selection criteria**

| Criteria | Inclusion | Exclusion |
| --- | --- | --- |
| Publication Type | Peer-reviewed | Not peer-reviewed (i.e., grey literature) |
| Publication Access | Full text available through a library service | Full-text not accessible |
| Language | English | Non-English |
| Study Design/Type | Clinical studies, including randomized/non-randomized designs (e.g., randomized controlled trials, observational studies) | Animal studies, non-clinical trials, reviews, opinions/editorials, ongoing clinical trials |
| Population | Adults; children | No population was excluded |
| Time Limit | Any time | None |
| Treatment | IFN, TLR, IFN-stimulated gene | Not IFN, TLR, IFN-stimulated gene, TLR used as an adjuvant for vaccines |
| Disease Area | Any disease area with potential relevance for the research questions | Diseases in which IFN was given for a non-respiratory chronic condition (i.e., Hepatitis C, Multiple sclerosis) |
