# Supplementary material for: Interferon-based agents for current and future viral respiratory infections: A scoping literature review of human studies
Source: PLOS Glob Public Health. 2022 Apr 6;2(4):e0000231. doi: 10.1371/journal.pgph.0000231 (PMC10022196; doi:10.1371/journal.pgph.0000231)
Supplement: S4 Table — (DOCX) [file pgph.0000231.s004.docx]

**SI Table 4: Data extracted from articles**

| Category | Data Extracted |
| --- | --- |
| Article Information | Title; authors; journal; publication year; DOI; language; type of study/data |
| Methods | Treatment category (IFN, TLR, or IFN-stimulated gene); pathogen (if applicable); drug; population; disease severity; timing of drug administration; mode of drug administration; pathway involved |
| Outcomes | Efficacy; tolerability; safety; toxicity; side effects; complications; adverse reactions; patient stratification biomarkers; pharmacodynamic markers; pharmacokinetics; genomics; transcriptomics; systems virology; and inflammation |
