# Supplementary material for: Interferon-based agents for current and future viral respiratory infections: A scoping literature review of human studies
Source: PLOS Glob Public Health. 2022 Apr 6;2(4):e0000231. doi: 10.1371/journal.pgph.0000231 (PMC10022196; doi:10.1371/journal.pgph.0000231)
Supplement: Attachment — Submitted filename: Response to reviewers_30_1_2022_to_submit.pdf [file pgph.0000231.s005.pdf]

UNIVERSITY of WASHINGTON
DEPARTMENT OF GLOBAL HEALTH
SCHOOL OF MEDICINE & SCHOOL OF PUBLIC HEALTH

January 30, 2022

Dear Dr. Asaduzzaman,

Enclosed please find the revised manuscript, entitled "Interferon-based agents for current and future viral respiratory infections: a scoping literature review of human studies" for consideration in *PLOS Global Public Health.*

We appreciate the reviewers' thoughtful comments and have addressed each comment in the marked-up manuscript (with yellow highlight), and below in the tables, by reviewer, on pages 1-10.

As you will appreciate, most of the reviewer comments regarded added clarity on our reporting of our methods and results. We have successfully addressed each point and believe this manuscript to be a stronger contribution to the literature than our initial submission due to the added clarity and transparency from this peer review process.

Thank you for the opportunity to re-submit our manuscript to *PLOS Global Public Health.*

Gratefully,

Aldina Mesic, MPH

Ninth and Jefferson Building Box 359931 325 9th Avenue Seattle, WA 98104-2499 206.221.4970

deptgh@uw.edu globalhealth.washington.edu

Table 1: Reviewer 1's Comments and Responses

**Reviewer's Comment**	**Response**
I appreciate the effort of the authors for the interesting article. The article is well written, but there are some methodological disparities. There is scope for improvement. Please find my comments here:	Thank you very much for your thoughtful feedback. We have addressed your comments below, and hope that you will agree that the write up now meets top standards for methodological conduct and reporting.
As per the PRISMA-ScR checklist, the protocol should be registered. Did the author register the protocol?	We have registered the protocol on Open Science Framework. The DOI is here: 10.17605/OSF.IO/9G3SH.<br><br>We have updated this information in the Methods section on page 5, lines 109-111 and in the Supplementary Materials on page 1.
Study Selection (Page 4): The title and abstract screening should be conducted by two independent review authors. It is not mentioned. Did the authors screen independently?	Yes, three authors (AM, EJ, ML) screened titles and abstracts independently.<br><br>This information has been clarified in the Methods section on page 6, line 153.
Study Selection (Page 4): The full text screening should be conducted by two independent review authors. It is not mentioned. Did the authors screen independently? If independent screening is not done, it should be mentioned in the limitation.	Yes, three authors (AM, EJ, ML) were involved with the full text screening. A primary review was conducted by one of two authors (EJ or ML) and then a secondary review was conducted by the lead author (AM). Thus, full text screening was conducted by two independent review authors for each article.<br><br>This information has been added to the Methods section on page 6, lines 161-162.
Ideally, the data extraction should be conducted by two independent review authors. Please mention if not done so.	Four authors were involved in the overall data extraction process. One of two authors did the first data extraction (EJ or ML), and the lead author (AM) conducted the second data extraction. Four of the co-authors reviewed the data extraction results (AM, EJ, ML, RP).<br><br>We have clarified this in the Methods section on page 6, lines 166-167 and 175-176.
In inclusion criteria, the authors have mentioned that only peer reviewed articles were included. What was the logic for searching medRxiv? Is medRxiv a database for peer reviewed articles?	We included articles from medRxiv as the literature on interferon and COVID-19 was evolving so rapidly that relying on only databases for published studies would miss some important articles assessing the impact of interferon-based agents on COVID-19.<br><br>We have clarified that we included peer-reviewed articles and pre-prints (defined as unpublished manuscripts that are in the

	process of peer review) in the Methods section on page 5, lines 139-143.
As per PRISMA-ScR checklist, quality assessment should be conducted. Authors might use the JBI quality assessment checklist for RCTs and other study designs.	Thank you for this suggestion. We opted not to conduct a critical appraisal of the literature, which is considered optional as part of a scoping review, whereas it is required as part of a systematic review. For instance, Tricco et al. state *"Critical appraisal of individual sources of evidence falls outside the realm of scoping review methodology (4, 5), it may be done if relevant to the scoping review objectives. […] Because this step is optional, authors are expected to report the results only if an appraisal was done."* (Tricco et al., 2018).<br><br>We decided not to critically appraise the literature as the intention of our work was not to appraise, for example for quality of the study design, or synthesize, for example for derivation of meta-analytic point estimates, the studies we examined. Rather, our goal was to understand the scope and coverage of the body of literature for interferons and viral respiratory infections.

Table 2: Reviewer 2's Comments and Responses

Lines 349-350: "While this is one of the first scoping reviews to examine the existing literature on IFN-based agents on viral respiratory infections, it has several limitations." Could you add the reference of the existing scoping reviews and also compare similarities and differences with your scoping review, which I guess is the most updated and it would be interesting to know, what your review adds to the existing ones.	We have not seen another systematic or scoping review which has explored the safety and efficacy of IFN-based agents (IFN, IFN-stimulating agents, toll-like receptor agonists) on viral respiratory infections. The aim of our work was to try to understand how interferon may work for currently recognized viral infections, as well as future unrecognized pathogens, rather than focusing on one disease. Further, we wanted to understand the efficacy of IFN-base agents as pre-exposure prophylaxis, post-exposure prophylaxis, and treatment.<br><br>Most other systematic and scoping reviews have explored IFN-based agents for the treatment of diseases such as hepatitis C, cancer, Behcet's syndrome, multiple sclerosis, and others. Many other reviews (published and ongoing) have assessed the role of interferon for COVID-19 (Nakhlband et al., 2021; Sosa et al., 2021; Walz et al., 2021). Some examples of published work and ongoing reviews are listed below:<br><br>Published:<br>• Sosa JP, Caceres MM, Comptis JR, Quiros J, Príncipe-Meneses FS, Riva-Moscoso A, Belizaire MP, Malanyaon FQ, Agadi K, Jaffery SS, Sahajwani J. Effects of interferon beta in COVID-19 adult patients: systematic review. Infection & chemotherapy. 2021 Jun;53(2):247.<br>• Walz L, Cohen AJ, Rebaza AP, Vanchieri J, SladeMD, Cruz CS, Sharma L. JAK-inhibitor and type I interferon ability to produce favorable clinical outcomes in COVID-19 patients: a systematic review and meta-analysis. BMC infectious diseases. 2021 Dec;21(1):1-0.<br>• Nakhlband A, Fakhari A, Azizi H. Interferon-beta offers promising avenues to COVID-19 treatment: a systematic review and meta-analysis of clinical trial studies. Naunyn-schmiedeberg's Archives of Pharmacology. 2021 Feb 15:1-0.<br><br>Ongoing systematic reviews:<br>• Interferon to treat COVID-19<br>• Interferon-beta 1b to treat COVID-19

	• Janus Kinase Inhibitor and Type I Interferon to treat COVID-19<br>• Interferon-alpha to treat COVID-19<br>• Interferon on non-severe COVID-19<br><br>The systematic review which has the greatest similarity to our work is the following study which focuses on COVID-19, MERS, and SARS (Saleki et al., 2021). However, it only covered three pathogens, focused on treatment, and reviewed grey literature (i.e., not peer reviewed studies such as case reports) and peer-reviewed literature, which is notable different from our methods.<br><br>• Saleki K, Yaribash S, Banazadeh M, Hajihosseinlou E, Gouravani M, Saghazadeh A, Rezaei N. Interferon therapy in patients with SARS, MERS, and COVID-19: A systematic review and meta-analysis of clinical studies. European journal of pharmacology. 2021 Jun 12:174248.<br><br>We have expanded on the prior literature, including key similarities and differences in the Introduction section on page 3, lines 91-99.
There are many systematic reviews (SRs) addressing interventions for COVID-19, including interferon-based therapies. Do your results agree with these SRs?	Our results did agree with several systematic reviews which have been conducted for COVID-19, including those aforementioned (Nakhlband et al., 2021; Saleki et al., 2021; Sosa et al., 2021; Walz et al., 2021).<br><br>Specifically, Saleki *et al.* evaluated the clinical efficacy of IFN-based treatments and combination therapy with antivirals, corticosteroids, traditional medicine, and other treatments for SARS, MERS, and COVID-19. It was shown that combination therapy of IFN with antivirals (favipiravir, arbidol, remdesivir) and corticosteroids had benefits such as chest X-ray improvement and lower levels of inflammatory cytokines. The authors noted a lack of high-quality cohorts and controlled trials (Saleki et al., 2021).<br><br>Another study found that Type 1 interferons were associated with mortality and hospital discharge. However, they similarly noted a need for additional well-designed randomized controlled trials (Walz et al., 2021).

	Nakhlband *et al.* concluded that more extensive interventions need to be studied in patients (Nakhlband et al., 2021). Sosa *et al* found IFN-β has been shown to decrease the hospitalization's length and decrease the severity of respiratory symptoms with few side effects but similarly called for additional randomized controlled trials (Sosa et al., 2021).<br><br>We have included this updated information in the Discussion on page 18, lines 352-357 and 374-375.
The authors did not restrict the search to adults only. Where results stratified by age? Where there any studies on children?	This is correct, we did not restrict the search to adults. One study included infants aged 0-12 months with bronchiolitis (Chen et al., 2020).<br><br>We appreciate the reviewer raising this important point. We have now clarified this in *Table 1*, and in the Methods on page 6, lines 156-157, and in the Results on page 6, lines 190-191. We have discussed this study separately given the different population (page 14, lines 299-302).
Literature search:<br>Lines 124-125: The authors conducted the searches in October and December 2020 and February 2021. Why did you skip the months of November 2020 and January 2021? Or did you mean from October 2020 to February 2021?	Thank you for pointing out this lack of clarity. We conducted searches from October 2020 to February 2021, repeating the same search every 4 weeks to capture new studies as they were published.<br><br>We have corrected this sentence in the Methods section on page 5, lines 135-137.
Line 128 : The authors included human clinical trials. However, in the Supplementary Materials 3: Study selection criteria table they included also observational studies. Although in the Supplementary Materials 2: Search terms table they only added search terms for clinical trials and not for observational studies "(Clinical Trial[sb] OR Multicenter Study[sb] OR "Clinical Trials as Topic"[Mesh] OR "Multicenter Studies as Topic"[Mesh")	Thank you for highlighting this discrepancy. Initially, we had set out to only capture clinical trials (including phase I-IV, multi-center studies).<br><br>However, three of the studies (Pereda et al., 2020; Wang et al., 2020) that focused on COVID-19 were obtained from medRxiv, where we could not filter by clinical trial. However, given the importance of the findings and relevance to the ongoing pandemic, we felt as if they should be included given that many clinical trials were not reporting results yet.<br><br>We have clarified this in the Results section on page 6, lines 188-190. We have also updated the title to say

	'human studies' rather than 'human interventional trials' for greater clarity.
In general, please clarify the target population. Are children and adults included ? I do not know about scoping reviews but of systematic review, still I think it would be good to have the P of the PECOS.	We did not specify a target population and included studies which recruited healthy adults (for pre-exposure prophylaxis, post-exposure prophylaxis) and adults and children for treatment.<br><br>We have added a section for population in *Table 1* and described the findings in the Results section on page 6, lines 190-191. We have also now included a "Population Column" in *Tables 2-4.*
Results:<br>Lines 158-164: Please check the first paragraph related to Figure 1. The numbers given do not quite tally. Please correct. Figure 1 : There are several mistakes listed here:<br>Abstracts screened: 977 and excluded 744. Thus, full text articles assessed for eligibility should be 233, or excluded should be 783 if the full articles assessed were truly 194...Please correct<br>Records excluded during full text review do not add up to 146 but to 159. Please correct.	Thank you for pointing this out and we greatly apologize for any errors on our part. We have updated the numbers to accurately reflect the numbers of studies found, reviewed, and including.<br><br>This information is updated in the Results on page 6, lines 180-186 and in *Figure 1.*
Table 1: It would be good to also have the info on the country these studies were published, to have an idea where this topic is relevant, and also it will give us indirect information on ethnicity (efficacy of treatment may vary among different ethnic groups, maybe this explains variability in efficacy).	Thank you for this important suggestion.<br><br>We have now added a section on country where the study took place in *Table* 1, summarized the countries in the Results section on page 6-7, lines 191-192, and described the countries for each study in *Tables 2-4.*
Tables in general : In those observational studies, how where participants recruited. Where there recruited from the general population, from hospitals etc.	There were two observational studies focused on COVID-19 (Pereda et al., 2020; Wang et al., 2020).<br><br>We have now included information about the study population for each study in *Tables 2-4.*
Line 166: The authors found 16 studies published between 1981 and 1990. If the search was conducted in 2020-2021 one would expect to only retrieve those articles published in this period of time. I think what has happened here is a case of a misleading report: maybe the authors conducted a search from inception until February 2021 but did not report it appropriately? It is difficult to	We apologize for the lack of clarity on this point. Our search covered all articles from inception to the time that the study ended (February 2021). We did not want to exclude any older studies (i.e., from the 1970s) when interferon was discovered and of high scientific interest. When our initial search was conducted (October 2020), we captured all articles from before that date, and re-ran the search every 4 weeks to look specifically for

understand when you truly conducted the search. You may have conducted it initially from inception to October 2020, then updated it until December 2020 and then further expanded covering until February 2021. (lines 366-369 you finally explained what I understood... that you re-ran the searches several times. Still you have to make it clear what your search covers.)	COVID-19 studies, or other studies that may have been published from October 2020-February 2021.<br><br>We clarified this point in the Methods section on page 5, lines 135-137.
Results are reported per type of IFN. Since there are no subheadings I think for the reader it would be great if the authors start with IFN-alpha they continue to use the same terminology. For example, in lines between 181 to 196 one can read: IFN- a, IFN, and IFN-a. It also applies to other types: IFN- §, IFN-B etc. Please keep one at a time. IFN when general, IFN-a when discussing results related to alpha and the same for the rest of the types. It is in general applicable to all sections within results.	We appreciate this point and have now added sub-headings and we have updated the wording as appropriate throughout the results section to clarify which type of IFN was being studied.
Table 2: Regarding the study design, some are labelled as randomized and others have no information on randomization. Given that you only included RCTs and observational studies, either you add the term in the rest of the studies or you mention it more clearly in the inclusion criteria in the text (line 128). There is one study that is longitudinal, open-label. Is it randomized, or is it an observational study? It would be good to differentiate both type of studies. Could you standardize the information provided e.g. :<br>Randomized<br>Parallel or cross-over etc<br>Type of blinding (open label, single- or double-blind, or observer-blind) Placebo-controlled<br>Challenge<br>Observational (type of design: cross-sectional, cohort, case-control etc)	This is an important point as it would help readers quickly ascertain the study design. We had initially used the same terminology as the authors of the studies did for the "study type" column.<br><br>To aide in understanding and make the categories more consistent, we have added a section for "Randomization", "Blinding", "Placebo", and "Challenge" in *Table 1*.<br><br>We have also added detailed information on the study design for each study in *Tables 2-4.*
Treatment Results:<br>Line 239: 7 studies used IFN-a. However only 6 references are provided in the text: 43, 45-49.	Apologies for our error here, and this has now been updated in the Results section on page 9.

Line 266: Hung et al. has no reference number in the text.	Apologies for our error here, and this has now been updated in the Results section on page 14, line 314.
Line 268: Monk et al has no reference number in the text.	Apologies for our error here, and this has now been updated in the Results section on page 14, line 315.
Line 276: Put the one administering IFN-λ in a separate paragraph, otherwise you think it is a study administering IFN-κ Just to avoid confusion and following the pattern you've been using throughout the manuscript.	This is a helpful suggestion, and we have now added sub headers and separated the paragraphs on pages 9-17.
Table 4: You have included non-randomized trials but according to the Supplementary Materials 3: Study selection criteria table you included randomized clinical trials. This deserves some clarification. What were the inclusion and exclusion criteria regarding the study type then?	Please see comment and response above for reviewer 1, and we apologize for this miscommunication on our part. We wanted to include all clinical trials with interferon (randomized and otherwise) and include additional observational studies if they were focused on COVID-19 and found on medRxiv, where we could not filter by "clinical trial".<br><br>We have clarified this in the *Supplementary Materials 3: Study selection criteria.*
In the results section it should be described how many studies were of clinical design and how many were of observational design since the strength of the evidence will be different. To assess efficacy and safety, clinical trials are recommended over observational studies.	We agree with this point, also raised by reviewer 1, and have updated *Table 1* to include a section on observational vs. clinical trial design and have described this in the Results section on page 6, lines 189-190.
Lines 305 and 311: Could you please add the references of the studies that commented on the pharmacokinetic and pharmacodynamic outcome data? And also indicate the type of IFN administered?	To the best of our knowledge, only three studies discussed pharmacokinetic/dynamic outcomes (Farr et al., 1984; Samo et al., 1983; Sperber et al., 1988). We decided not to focus on this in the results given the scarcity of studies which covered these topics.<br><br>We had added information about the studies in the Discussion on 18, lines 366-367.

References for Response:

Chen, L., Shi, M., Deng, Q., Liu, W., Li, Q., Ye, P., Yu, X., Zhang, B., Xu, Y., Li, X., Yang, Y., Li, M., Yan, Y., Xu, Z., Yu, J., Xiang, L., Tang, X., Wan, G., Cai, Q., . . . Liu, H. (2020). A multi-center randomized prospective study on the treatment of infant bronchiolitis with interferon α1b nebulization. *PLoS One*, *15*(2), e0228391. https://doi.org/10.1371/journal.pone.0228391

Farr, B. M., Gwaltney, J. M., Jr., Adams, K. F., & Hayden, F. G. (1984). Intranasal interferon-alpha 2 for prevention of natural rhinovirus colds. *Antimicrob Agents Chemother*, *26*(1), 31-34. https://doi.org/10.1128/aac.26.1.31

Nakhlband, A., Fakhari, A., & Azizi, H. (2021). Interferon-beta offers promising avenues to COVID-19 treatment: a systematic review and meta-analysis of clinical trial studies. *Naunyn-schmiedeberg's Archives of Pharmacology*, 1-10.

Pereda, R., González, D., Rivero, H. B., Rivero, J. C., Pérez, A., López, L. D. R., Mezquia, N., Venegas, R., Betancourt, J. R., & Domínguez, R. E. (2020). Therapeutic Effectiveness of Interferon-α2b Against COVID-19: The Cuban Experience. *J Interferon Cytokine Res*, *40*(9), 438-442. https://doi.org/10.1089/jir.2020.0124

Saleki, K., Yaribash, S., Banazadeh, M., Hajihosseinlou, E., Gouravani, M., Saghazadeh, A., & Rezaei, N. (2021). Interferon therapy in patients with SARS, MERS, and COVID-19: A systematic review and meta-analysis of clinical studies. *European journal of pharmacology*, 174248.

Samo, T. C., Greenberg, S. B., Couch, R. B., Quarles, J., Johnson, P. E., Hook, S., & Harmon, M. W. (1983). Efficacy and tolerance of intranasally applied recombinant leukocyte A interferon in normal volunteers. *J Infect Dis*, *148*(3), 535-542. https://doi.org/10.1093/infdis/148.3.535

Sosa, J. P., Caceres, M. M. F., Comptis, J. R., Quiros, J., Príncipe-Meneses, F. S., Riva-Moscoso, A., Belizaire, M.-P., Malanyaon, F. Q., Agadi, K., & Jaffery, S. S. (2021). Effects of interferon beta in COVID-19 adult patients: systematic review. *Infection & chemotherapy*, *53*(2), 247.

Sperber, S. J., Levine, P. A., Innes, D. J., Mills, S. E., & Hayden, F. G. (1988). Tolerance and efficacy of intranasal administration of recombinant beta serine interferon in healthy adults. *J Infect Dis*, *158*(1), 166-175. https://doi.org/10.1093/infdis/158.1.166

Tricco, A. C., Lillie, E., Zarin, W., O'Brien, K. K., Colquhoun, H., Levac, D., Moher, D., Peters, M. D. J., Horsley, T., Weeks, L., Hempel, S., Akl, E. A., Chang, C., McGowan, J., Stewart, L., Hartling, L., Aldcroft, A., Wilson, M. G., Garritty, C., . . . Straus, S. E. (2018). PRISMA Extension for Scoping Reviews (PRISMA-ScR): Checklist and Explanation. *Ann Intern Med*, *169*(7), 467-473. https://doi.org/10.7326/m18-0850

Walz, L., Cohen, A. J., Rebaza, A. P., Vanchieri, J., Slade, M. D., Cruz, C. S. D., & Sharma, L. (2021). JAK-inhibitor and type I interferon ability to produce favorable clinical outcomes in COVID-19 patients: a systematic review and meta-analysis. *BMC infectious diseases*, *21*(1), 1-10.

Wang, N., Zhan, Y., Zhu, L., Hou, Z., Liu, F., Song, P., Qiu, F., Wang, X., Zou, X., Wan, D., Qian, X., Wang, S., Guo, Y., Yu, H., Cui, M., Tong, G., Xu, Y., Zheng, Z., Lu, Y., & Hong, P. (2020). Retrospective Multicenter Cohort Study Shows Early Interferon Therapy Is Associated with Favorable Clinical Responses in COVID-19 Patients. *Cell Host Microbe*, *28*(3), 455-464.e452. https://doi.org/10.1016/j.chom.2020.07.005
